# Interactions among poverty, gender, and health systems affect women’s participation in services to prevent HIV transmission from mother to child: A causal loop analysis

**DOI:** 10.1371/journal.pone.0197239

**Published:** 2018-05-18

**Authors:** Jennifer Yourkavitch, Kristen Hassmiller Lich, Valerie L. Flax, Elialilia S. Okello, John Kadzandira, Anne Ruhweza Katahoire, Alister C. Munthali, James C. Thomas

**Affiliations:** 1 MEASURE Evaluation, Carolina Population Center, University of North Carolina, Chapel Hill, North Carolina, United States of America; 2 ICF, Rockville, Maryland, United States of America; 3 Department of Health Policy and Management, Gillings School of Global Public Health, University of North Carolina, Chapel Hill, North Carolina, United States of America; 4 RTI International, Durham, North Carolina, United States of America; 5 Department of Psychiatry, Makerere University, Kampala, Uganda; 6 Child Health and Development Centre, Makerere University, Kampala, Uganda; 7 Centre for Social Research, Chancellor College, University of Malawi, Zomba, Malawi; 8 Department of Epidemiology, Gillings School of Global Public Health, University of North Carolina, Chapel Hill, North Carolina, United States of America; The Ohio State University, UNITED STATES

## Abstract

Retention in care remains an important issue for prevention of mother-to-child transmission (PMTCT) programs according to WHO guidelines, formerly called the “Option B+” approach. The objective of this study was to examine how poverty, gender, and health system factors interact to influence women’s participation in PMTCT services. We used qualitative research, literature, and hypothesized variable connections to diagram causes and effects in causal loop models. We found that many factors, including antiretroviral therapy (ART) use, service design and quality, stigma, disclosure, spouse/partner influence, decision-making autonomy, and knowledge about PMTCT, influence psychosocial health, which in turn affects women’s participation in PMTCT services. Thus, interventions to improve psychosocial health need to address many factors to be successful. We also found that the design of PMTCT services, a modifiable factor, is important because it affects several other factors. We identified 66 feedback loops that may contribute to policy resistance—that is, a policy’s failure to have its intended effect. Our findings point to the need for a multipronged intervention to encourage women’s continued participation in PMTCT services and for longitudinal research to quantify and test our causal loop model.

## Introduction

Regular use of antiretroviral therapy (ART) and adherence to infant feeding guidelines maximize the likelihood of exposed infants’ HIV-free survival in resource-limited settings [1]. However, maternal, interpersonal, gender, and health system factors hinder implementation of the World Health Organization’s prevention of mother-to-child transmission (PMTCT) guidelines in sub-Saharan African countries, and further constrain health benefits for mothers and children [2,3]. WHO advises that all HIV-positive pregnant and breastfeeding women start triple ART as soon as they are diagnosed and continue treatment, regardless of their CD4 count, for the rest of their lives, an approach formerly called “Option B+” [4]. Some women participate in PMTCT programs but later discontinue during pregnancy or after their children are born [2]. Women who stop treatment are of particular concern, because their viral load is not suppressed and their children are at risk of HIV infection.

Multiple reasons for women’s discontinuing PMTCT programs have been documented; however, most studies on this topic pre-date Option B+. We and others have found that women have concerns about disclosure, stigma, optimal infant feeding, behavior of health workers, distance to facilities, and side effects of treatment. Having an HIV-free infant appears to have been a main motivation for participation, particularly among women who had discontinued the program [2,3,5–10]. In addition, some experts have voiced concerns about Option B+ that are related to gender factors [11]. In studies we conducted in Malawi and Uganda, we found that gender-related factors contribute to women’s discontinuation and also interact with poverty and health system factors to affect their participation [12,13].

Although the barriers to PMTCT participation are well documented, it is unclear how gender-related factors interact with other contextual factors to affect women’s participation under Option B+. The purpose of this study was to hypothesize the mechanisms through which all these factors are interconnected. We understand much about these factors individually, but less about how they contribute to the complexity of women’s lives; a better understanding of that complexity is needed to design effective interventions. Health programs often invest in shortsighted policies and experience policy resistance (the failure of policies to have their intended effects) in the face of complexity [14]. Knowing that those factors affect women’s participation is not enough; we must understand the complex system in which HIV-positive women live. System dynamics tools facilitate the understanding of complex mechanisms and create theories of change to inform decision making [15].

## Materials and methods

### Overview

We used causal loop diagramming to document the complexity among determinants of women’s PMTCT program participation. The causal loop diagram (CLD) integrates the complexities revealed by our previous research [2], a review of relevant literature, and our research team’s hypotheses about missing connections (Table 1).

**Table 1:** Factors affecting women’s participation in PMTCT programs under Option B+ policies.

Theme	Factors	No. arrows in	No. arrows out	Effects on other factors and on program participation (from literature, parent study findings, and hypothesized connections)
**Poverty**	Poverty	2	4	Lack of transportation to services (women [2,3] and men*); work [3,13]; food insecurity [2]; limited education*
	Food insecurity	1	1	Inability to take medication [2]; inability to feed infants according to guidance [2]; nutritional deficiencies and poor health*
	Limited education	1	2	Lack of knowledge about PMTCT*
	Knowledge about PMTCT	2	2	Perception that PMTCT is more for child than for mother [16]; psychosocial health*; participation [12,17]; service design (specifically, not understanding the education component [3], or education emphasizes child health and not maternal health*)
	ART use	5	3	ART regimen (begin immediately, take at same time each day, lifelong) leads to indirect status disclosure [3]; reluctance to start medication when feeling well [13,18,19]; side effects and fear of side effects [3,18,19]; domestic discord or fear of it (resulting from status disclosure or partner resentment if he does not have access to medication) [11,13,18,19] Psychosocial health*; physical health (side effects or improved health overall) [2,19]; participation [2]
	Physical health	1	2	Feel well [13,18]; have energy [13]; have an HIV-negative baby [2,18]; psychosocial health*; Work [19]; family responsibilities*; participation (could stop participating because they feel well* or because they feel unwell [3])
	Transportation	1	1	Ability to collect drugs and attend appointments [2]
	Psychosocial health	9	2	Disclosure (low self-esteem; domestic discord; lack of social support for mother; gossip; avoidance; discrimination)[2]; participation [2] Fear of stigma leads to medication adherence because one doesn’t want appearance to change*, but can also discourage PMTCT participation [2]
	Work	4	1	No time [2,19]; work location could affect continuity of care [3,13]; decision-making autonomy [13]; less poverty*
	No time	4	2	ART use [13,19]; participation [20]
**Gender**	Disclosure	3	2	Stigma [2]; psychosocial health [2]; domestic violence [2], verbal abuse [2], divorce [2], abandonment [2]; loss of economic support [2]; discovery of discordant couple status [2]; trying to participate in PMTCT program secretly [2]; partner support [2]
	Stigma	1	1	Psychosocial health [2]
	Decision-making autonomy	2	3	Work*; psychosocial health (self-efficacy; agency to participate and take medication; ability to negotiate safe sex)*; participation*
	Family responsibilities	0	2	No time*; work*; travel for caretaking responsibilities leads to discontinuity of care and disrupted ART use [2]
	Spouse/partner influence	0	3	ART use [2,18]; psychosocial health [2]; decision-making autonomy*
**Health system**	Service design	0	6	Service quality [2]; psychosocial health (inadequate time for counseling [2]; test and initiation of treatment at first ANC visit [2]; men feel unwelcome and do not accompany women [2];) [2]; disclosure: lack of privacy [2] or, supportive*; no time (too much time spent at clinic) [2]; knowledge about PMTCT [2]; poor tracking system (loss of client continuity of care) [17]; distance to health services [13]; peer support at clinic [2]; participation [2]
	Service quality	1	4	Disclosure: lack of privacy [2] or, supportive*; psychosocial health (health worker attitude) [2,18]; no time (staff shortage) [2]; participation [2]
**Outcome**	Participation in PMTCT program	8	2	Psychosocial health [13]; ART use [13]

*Hypothesized connection

### Data

This study was approved by the Institutional Review Board of the University of North Carolina, Chapel Hill (#15–1454). The parent study’s methods are documented elsewhere [2,12,13]. Briefly, for that study, we conducted a literature review to inform interview guides that were used to conduct in-depth interviews with 32 women participating in PMTCT services in Malawi (average age 30) and Uganda (average age 27); 32 Malawian women (average age 30) and 16 Ugandan women (average age 27) who had discontinued participation in PMTCT services; 16 Malawian and 17 Ugandan health workers who provide PMTCT services in the public health systems; and six Malawian and eight Ugandan stakeholders working in organizations supporting HIV/AIDS services. We also conducted eight focus group discussions in each country with men from communities in the catchment areas of health facilities providing PMTCT services; 77 Malawian men (average age 33) and 73 Ugandan men participated (average age 38). More information about participants is available in the published study findings [2]. The Centre for Social Research, Chancellor College, University of Malawi and the Child Health and Development Centre, Makerere University, Uganda collected the data in four urban districts (Lilongwe and Blantyre, Malawi; Kampala and Mbarara, Uganda) and four rural districts (Dowa and Thyolo, Malawi; Masaka and Ntungamo, Uganda). Signed or thumb-printed informed consent was obtained from each participant. We asked respondents about HIV disclosure experiences, stigma and violence related to HIV, ART side effects, distance to service sites, social support, community perceptions of women and HIV, male involvement in PMTCT programs, women’s workload, why women discontinued participation, gender roles in families, and ways to improve service delivery. The question guides were included as supplemental files in our previous publication [2]. We used qualitative content analysis methods to conduct cross-group analyses. We developed codebooks for each type of respondent, using first deductive codes based on the question guides and then inductive codes that arose from the data. We grouped codes together into key themes and created data matrices to facilitate analysis. Analytic methods are described in detail in the publication about our study results [2].

### Causal loop diagramming

In the present analysis, we used a causal loop diagram to elucidate connections between factors that influence PMTCT participation. A CLD comprises variables that are important in determining a particular outcome over time—in this case, PMTCT participation among HIV-positive pregnant women and mothers. CLDs are characterized by these main attributes:

1. The variables are noun phrases that can have measurable quantitative or qualitative values [15]. Our CLDs include variables that emerged from our own research and from literature.
2. When a change in one variable triggers a change in another variable, we describe that as a causal linkage and diagram it with an arrow [15].
3. When the first variable is increased (or decreased), the direction of the change that it triggers in the second variable is depicted by putting an S (same direction) or O (opposite direction) on the arrow [15]. Dashed lines indicate that the first variable may trigger a change in the second variable in either direction.
4. A hash mark indicates that the causal loop plays out over a protracted time. For example, people’s experience of stigma or another’s stigmatizing or discriminatory act may have an effect on their psychosocial health within hours, in the form of feeling bad about themselves. However, ART use may have a protracted effect on their physical health, occurring weeks later.
5. When a series of causal linkages connect back to a variable earlier in the pathway (close the circle), they create a feedback loop [15]. Feedback loops have an important impact on outcomes. They either reinforce the earlier change or undermine (balance) it as they close the loop. Identifying this cycle is important because it happens repeatedly, driving exponential growth or decay.

### Creating the CLDs

We reviewed the published results of the parent study [2,12,13] and identified key themes or factors (in the form of variables) and causal linkages among them (Table 1). For example, we drew an arrow from “Spouse/partner influence” to “ART use” because our study supplied evidence for that connection: “He [my husband] reminds me to take my medication and to go to the clinic on time.”—Malawian woman who participates in the PMTCT programme [2]. We also added findings from literature to Table 1, looking for more evidence of causal linkages. We then hypothesized additional connections. Researchers often study associations between exposures and outcomes but seldom explain the pathways and complexity, which is the goal of this analysis.

After diagramming the connections using Vensim DSS software (Ventana Systems Incorporated, version 5.8b), we counted the number of arrows coming into and out of each variable (Table 1). The more arrows that come into a variable, the harder that factor is to modify, but a high volume of arrows signals a need to pay attention to that factor. Superficial interventions targeting those factors will be unsuccessful because those factors have multiple determinants. A high volume of arrows coming out of a variable indicates the importance of modifying that factor because it affects many other things.

We also specifically identified feedback loops through this analysis (Table 2). Feedback loops are important because they lead to exponential (more than linear) change and can explain policy resistance. When researchers study causal linkages piecewise, they cannot account for causal loops [15]. We further categorize the loops as balancing (the direction of the initial variable changes over time) or reinforcing (the direction of the initial variable stays the same over time).

**Table 2:** Feedback loops identified among poverty, gender, and health system factors that influence women’s participation in prevention of mother-to-child transmission of HIV services.

Loop No.	Label	Description	Balancing or reinforcing
1.	ART use	ART use →participation →ART use	Reinforcing
2.	ART use	ART use →psychosocial health →participation →ART use	Reinforcing
3.	ART use	ART use →physical health →participation →ART use	Reinforcing or Balancing*
4.	ART use	ART use →physical health →work →no time →ART use	Balancing
5.	ART use	ART use →physical health →work →decision-making autonomy →participation →ART use	Reinforcing or Balancing*
6.	ART use	ART use →physical health →work →poverty →transportation →ART use	Reinforcing or Balancing*
7.	ART use	ART use →physical health →work →no time →PMTCT participation →ART use	Reinforcing or Balancing*
8.	ART use	ART use →physical health →work →poverty →food insecurity →ART use	Reinforcing
9.	ART use	ART use →physical health →work →decision-making autonomy →psychosocial health →PMTCT participation →ART use	Reinforcing or Balancing*
10.	ART use	ART use →physical health →work →poverty →limited education →knowledge about PMTCT →participation →ART use	Reinforcing or Balancing*
11.	ART use	ART use →physical health →work →poverty →limited education →knowledge about PMTCT →psychosocial health →participation →ART use	Reinforcing or Balancing*
12.	Poverty	Poverty →work →poverty	Balancing
13.	Poverty	Poverty →limited education →poverty	Reinforcing
14.	Poverty	Poverty →transportation →ART use →physical health →work →poverty	Reinforcing or Balancing*
15.	Poverty	Poverty →food insecurity →ART use →physical health →work →poverty	Reinforcing or Balancing*
16.	Poverty	Poverty →limited education →knowledge about PMTCT →participation →ART use →physical health →work →poverty	Reinforcing or Balancing*
17.	Poverty	Poverty →limited education →knowledge about PMTCT →psychosocial health →participation →ART use →physical health →work →poverty	Reinforcing or Balancing*
18.	Food insecurity	Food insecurity →ART use →physical health →work →poverty →food insecurity	Reinforcing or Balancing*
19.	Knowledge of PMTCT	Knowledge about PMTCT →participation →ART use →physical health →work →poverty →limited education →knowledge about PMTCT	Reinforcing or Balancing*
20.	Knowledge of PMTCT	Knowledge about PMTCT →psychosocial health →participation →ART use →physical health →work →poverty →limited education →knowledge about PMTCT	Reinforcing or Balancing*
21.	Limited education	Limited education →poverty →limited education	Reinforcing
22.	Limited education	Limited education →knowledge about PMTCT →participation →ART use →physical health →work →poverty →limited education	Reinforcing or Balancing*
23.	Limited education	Limited education →knowledge about PMTCT →psychosocial health →participation →ART use →physical health →work →poverty →limited education	Reinforcing or Balancing*
24.	Transportation	Transportation →ART use →physical health →work →poverty →transportation	Reinforcing or Balancing*
25.	Decision-making autonomy	Decision-making autonomy →work →decision-making autonomy	Reinforcing
26.	Decision-making autonomy	Decision-making autonomy →participation →ART use →physical health →work →decision-making autonomy	Reinforcing or Balancing*
27.	Decision-making autonomy	Decision-making autonomy →psychosocial health →participation →ART use →physical health →work →decision-making autonomy	Reinforcing or Balancing*
28.	PMTCT program participation	Participation →psychosocial health →participation	Reinforcing
29.	PMTCT program participation	Participation →ART use →participation	Reinforcing
30.	PMTCT program participation	Participation →ART use →psychosocial health →participation	Reinforcing
31.	PMTCT program participation	Participation →ART use →physical health →participation	Reinforcing or Balancing*
32.	PMTCT program participation	Participation →ART use →physical health →work →decision-making autonomy →participation	Reinforcing or Balancing*
33.	PMTCT program participation	Participation →ART use →physical health →work →no time →participation	Reinforcing or Balancing*
34.	PMTCT program participation	Participation →ART use →physical health →work →decision-making autonomy →psychosocial health →participation	Reinforcing or Balancing*
35.	PMTCT program participation	Participation →ART use →physical health →work →poverty →limited education →knowledge about PMTCT →participation	Reinforcing or Balancing*
36.	PMTCT program participation	Participation →ART use →physical health →work →poverty →limited education →knowledge about PMTCT →psychosocial health →participation	Reinforcing or Balancing*
37.	Physical health	Physical health →PMTCT participation →ART use →physical health	Reinforcing or Balancing*
38.	Physical health	Physical health →work →no time →ART use →physical health	Balancing
39.	Physical health	Physical health →work →poverty →transportation →ART use →physical health	Reinforcing or Balancing*
40.	Physical health	Physical health →work →poverty →food insecurity →ART use →physical health	Reinforcing or Balancing*
41.	Physical health	Physical health →work →no time →participation →ART use →physical health	Balancing
42.	Physical health	Physical health →work →decision-making autonomy →participation →ART use →physical health	Reinforcing or Balancing*
43.	Physical health	Physical health →work →decision-making autonomy →psychosocial health →participation →ART use →physical health	Reinforcing or Balancing*
44.	Physical health	Physical health →work →poverty →limited education →knowledge about PMTCT →participation →ART use →physical health	Reinforcing or Balancing*
45.	Physical health	Physical health →work →poverty →limited education →knowledge about PMTCT →psychosocial health →participation →ART use →physical health	Reinforcing or Balancing*
46.	Work	Work →poverty →work	Reinforcing
47.	Work	Work →decision-making autonomy →work	Reinforcing
48.	Work	Work →no time →ART use →physical health →work	Balancing
49.	Work	Work →no time →participation →ART use →physical health →work	Balancing
50.	Work	Work →poverty →food insecurity →ART use →physical health →work	Reinforcing or Balancing*
51.	Work	Work →decision-making autonomy →participation →ART use →physical health →work	Reinforcing or Balancing*
52.	Work	Work →poverty →transportation →ART use →physical health →work	Reinforcing or Balancing*
53.	Work	Work →decision-making autonomy → psychosocial health →participation →ART use →physical health →work	Reinforcing or Balancing*
54.	Work	Work →poverty →limited education →knowledge about PMTCT →participation →ART use →physical health →work	Reinforcing or Balancing*
55.	Work	Work →poverty →limited education →knowledge about PMTCT →psychosocial health →participation →ART use →physical health →work	Reinforcing or Balancing*
56.	Psychosocial health	Psychosocial health →participation →psychosocial health	Reinforcing
57.	Psychosocial health	Psychosocial health →disclosure →psychosocial health	Reinforcing
58.	Psychosocial health	Psychosocial health →participation →ART use →psychosocial health	Reinforcing
59.	Psychosocial health	Psychosocial health →disclosure →stigma →psychosocial health	Balancing
60.	Psychosocial health	Psychosocial health →participation →ART use →physical health →work →decision-making autonomy →psychosocial health	Reinforcing or Balancing*
61.	Psychosocial health	Psychosocial health →participation →ART use →physical health →work →poverty →limited education →knowledge about PMTCT →psychosocial health	Reinforcing or Balancing*
62.	Stigma	Stigma →psychosocial health →disclosure →stigma	Reinforcing
63.	Disclosure	Disclosure →psychosocial health →disclosure	Reinforcing
64.	Disclosure	Disclosure →stigma →psychosocial health →disclosure	Balancing
65.	No time	No time →ART use →physical health →work →no time	Reinforcing or Balancing*
66.	No time	No time →participation →ART use →physical health →work →no time	Reinforcing or Balancing*

*Dependent on ART effects on physical health; ART could improve health or unpleasant side effects could negatively affect physical health [19].

## Results

We documented the factors that influenced PMTCT program participation and their interrelationships in Table 1, using information from our published research and other literature, and hypothesized connections. Previous research indicated connections between poverty and lack of transportation to services; poverty and food insecurity; ART use and status disclosure; ART use and physical health; status disclosure and stigma; stigma and psychosocial health; spouse or partner influence and ART use; service quality and psychosocial health, among others (Table 1). Our published research with more than 270 informants confirmed many of these connections and also identified other gender- and service- related connections among key themes (Table 1).

**Fig 1:**
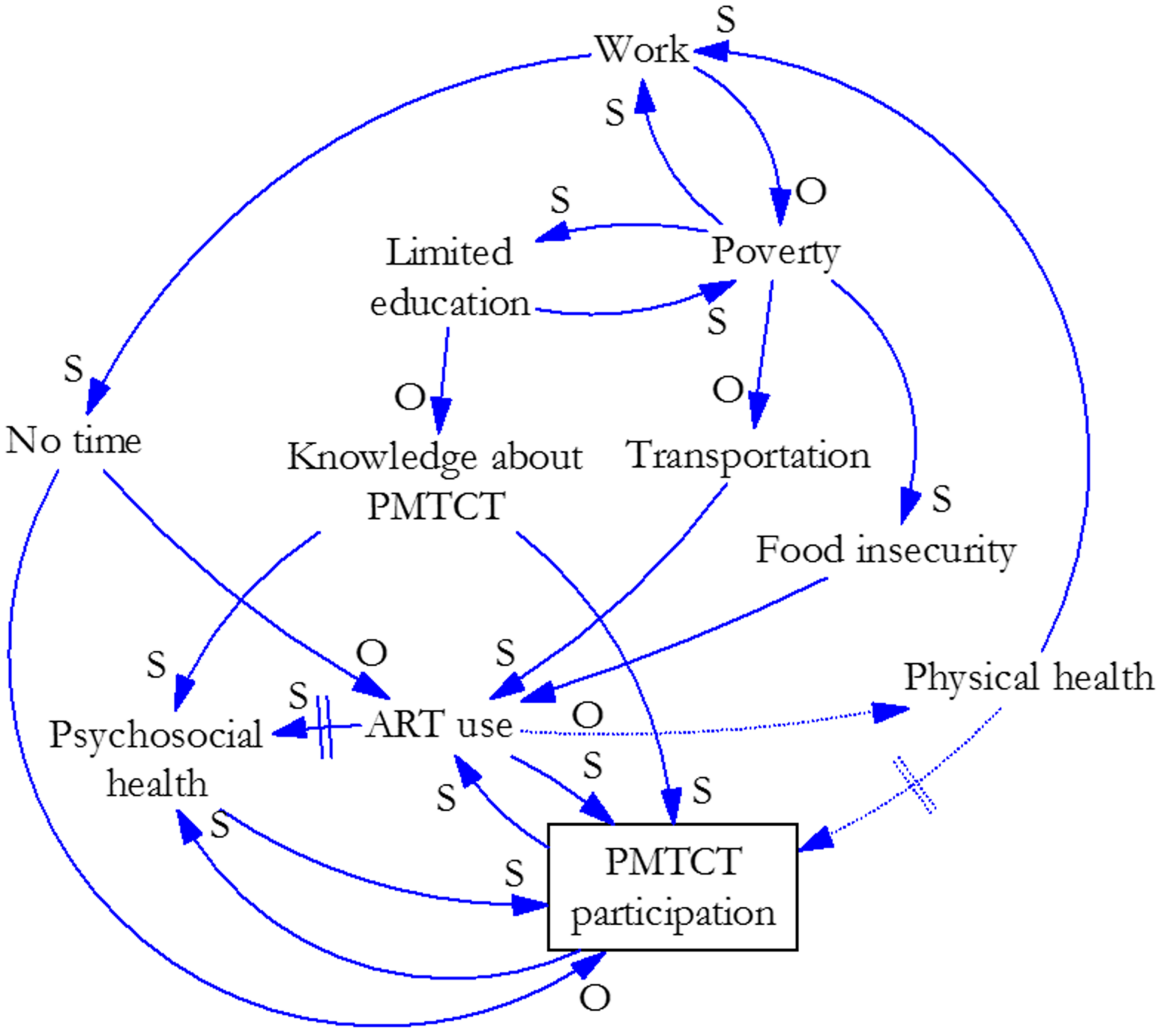
Poverty-related influences on women’s participation in PMTCT services.

**Fig 2:**
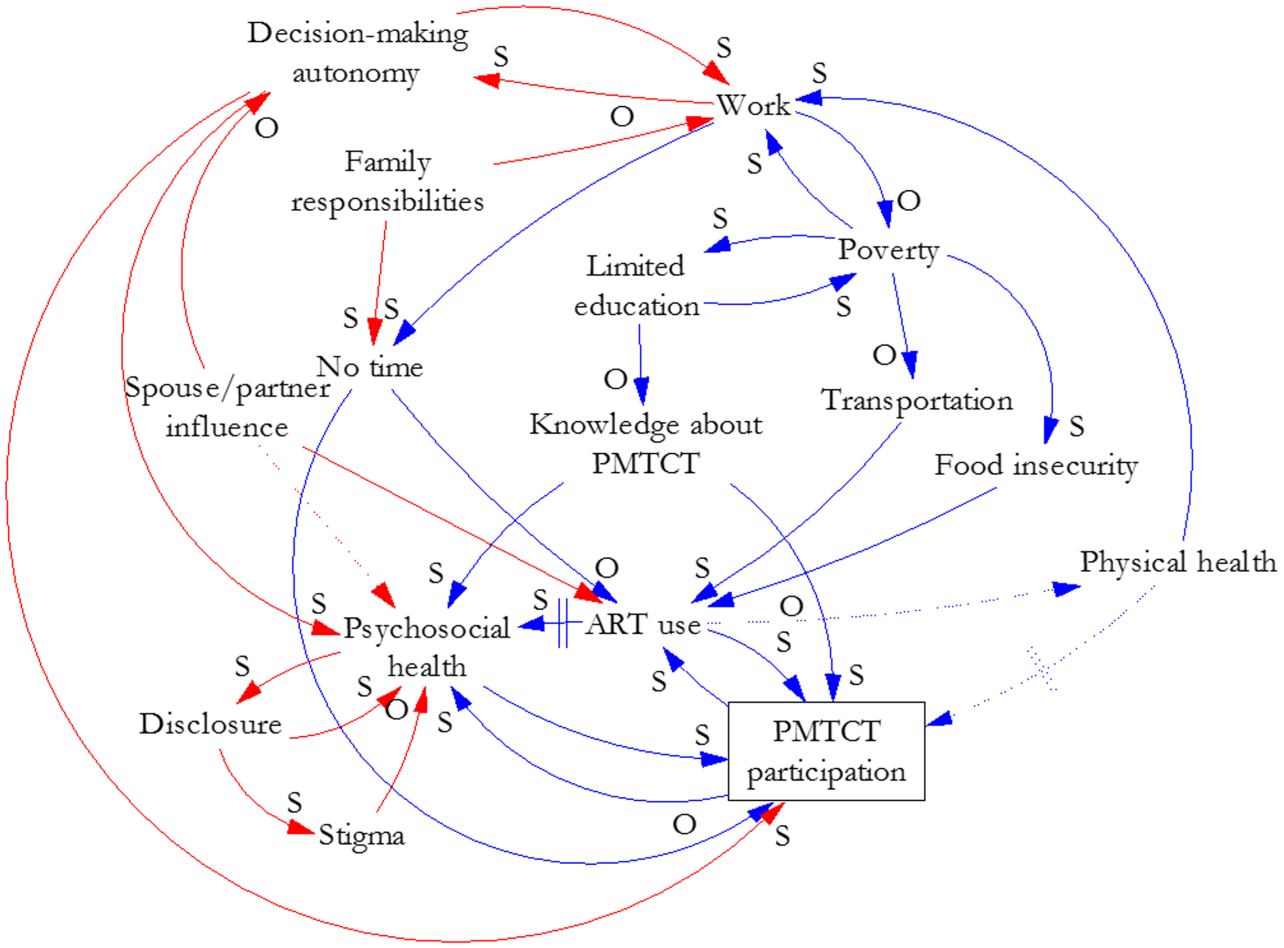
Gender and poverty-related influences on women’s participation in PMTCT services.

**Fig 3:**
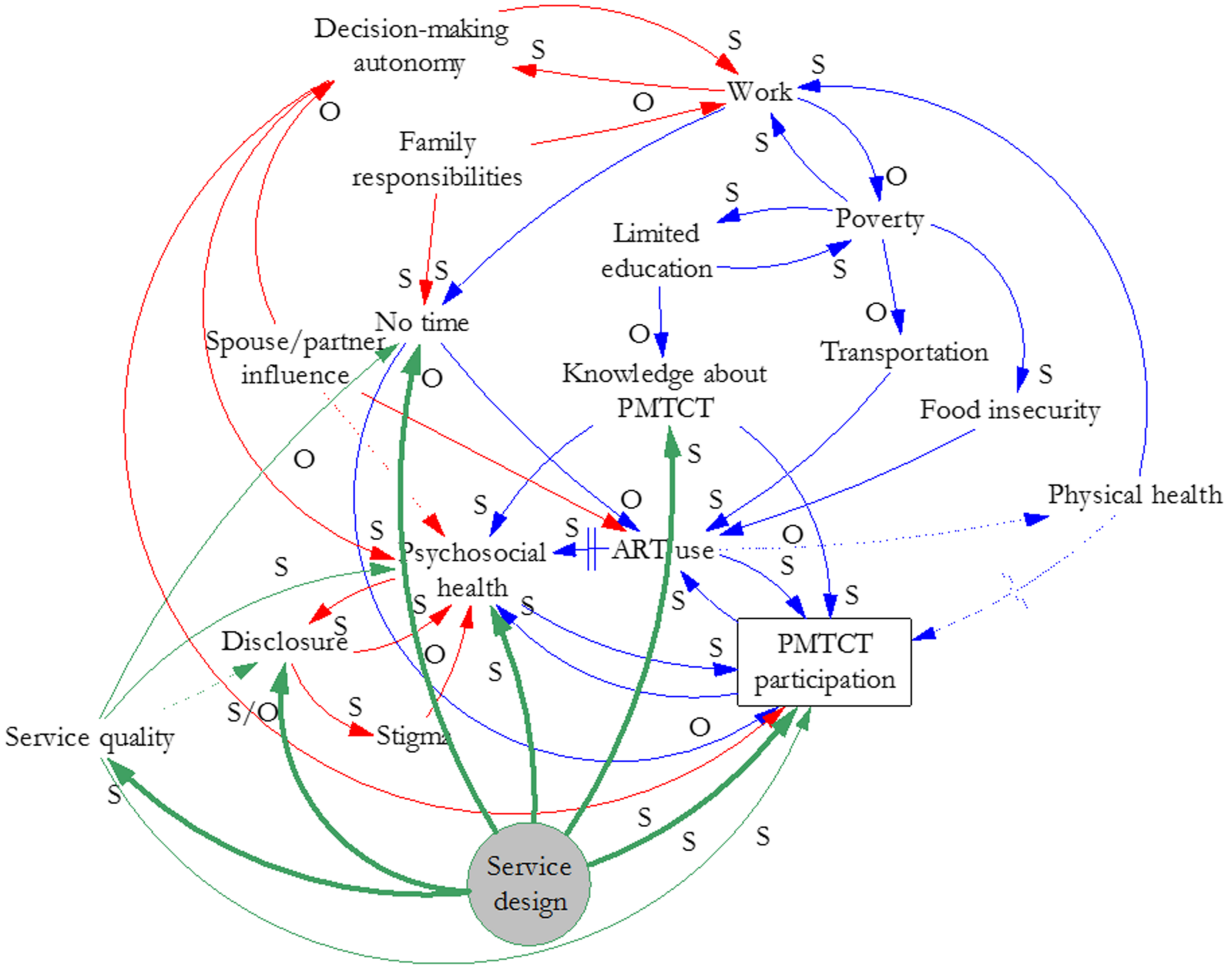
Health system, gender, and poverty-related influences on women’s participation in PMTCT services.

Our analysis and synthesis of this information yielded three causal loop diagrams that illustrate the effects of key factors and their interrelationships on women’s participation in PMTCT services. Fig 1 illustrates the factor group we labeled Poverty. These factors could apply to men or women and HIV services broadly, although we focused on women’s participation in PMTCT services for this study. Fig 2 overlays gender-related factors (red) and their interrelationships on the factors in Fig 1 to illustrate how gender interacts with poverty to create particular effects on women’s participation in PMTCT services. Fig 3 illustrates how elements of the health system (green) interact with gender and poverty to compound effects on women’s participation in PMTCT services. We describe the connections depicted in each figure below. The names of variables are underlined where they are the subject of the interrelationships presented.

### Poverty

Fig 1 shows that PMTCT service participation (defined as attendance at PMTCT services, which are usually based at facilities) is affected directly by knowledge about PMTCT, psychosocial health (defined as both mental and social well-being), ART use, lack of time, and physical health. Knowledge about PMTCT and services may motivate women to participate. If they do not understand the importance of the services and how to prevent HIV transmission to their children, they may be less likely to participate. Lack of knowledge about PMTCT may result from a limited education, which is determined by poverty, or from limited health education, determined by the service design (defined here as how PMTCT services are delivered, including infrastructure and type and number of human resources).

If a woman is depressed or has low self-esteem or self-efficacy, she could be less likely to obtain PMTCT services, as our research indicated [2]. Her psychosocial health is affected by her knowledge about PMTCT (and education and poverty) and her use of ART. When her psychosocial health is good (e.g., if she has social support from family members and accepts her condition or feels in control of her life), a woman may be more likely to engage with the health system by participating in PMTCT services and taking ART. Her psychosocial health may continue to improve if she feels better (from ART) and receives social support (from participation in PMTCT services through peers and health workers).

ART use contributes directly to psychosocial health, physical health, and PMTCT participation, and is directly affected by transportation, food insecurity, lack of time, and PMTCT participation. Women are often advised to take ART with food, and they cannot obtain the medication without transportation (when needed); both factors are determined by poverty.

Women need time to participate in PMTCT services in order to obtain ART and to take it regularly each day. Lack of time is affected directly by work, which is determined by poverty and physical health. Depending on the type of work a woman does, the more she works, the less time she may have to take ART and participate in PMTCT services. In many settings, the poorer she is, the more she may have to work. The better she feels, the more she can work. This creates a potentially disincentivizing cycle, or balancing loop. When she participates in PMTCT and takes ART, she will most likely experience psychosocial and physical health benefits, which can increase her ability to work (the need for which is determined by her poverty level). However, increased work may decrease the amount of time she has available to participate in PMTCT services and to take ART.

If her participation in PMTCT services decreases, so will her use of ART. Then her psychosocial and physical health will lessen over time, thereby impairing her ability to work and potentially deepening her poverty. Physical health gained from participation in PMTCT services may create an additional, and direct, disincentive to continue participating, because some women may not see a need to continue when they are feeling well. Furthermore, they may not initiate treatment if they feel well [19]. On the other hand, ART use may cause side effects that negatively affect physical health [19].

### Gender

Fig 2 incorporates gender-related factors (red) in the poverty-related relationships depicted in Fig 1. Work and decision-making autonomy are in a reinforcing loop: as work increases, autonomy increases, and vice versa. Decision-making autonomy affects psychosocial health and PMTCT program participation.

Spouses or partners influence autonomy, psychosocial health, and ART use. In some cases, senior family members also influence autonomy. That influence affects the work-autonomy relationship and can inhibit ART use and PMTCT program participation. Those actors also affect psychosocial health through social support or by being unsupportive.

Psychosocial health and HIV status disclosure are in a reinforcing loop. Whereas good psychosocial health can make it easier for a woman to disclose her status, poor psychosocial health may discourage disclosure. Disclosure may increase her psychosocial health if the person to whom she discloses is supportive, and that experience will facilitate her disclosure to others. However, if she and her partner have discordant status, particularly if she is HIV-positive, then her psychosocial health can be negatively affected. One health worker in Uganda said, “…where the man is the one who is negative and the woman is positive, this woman is going to be isolated. She is going to be depressed” [13]. Disclosure can lead to stigma, which negatively affects both psychosocial health and further disclosure. The effect on psychosocial health affects participation in the program.

A woman’s family responsibilities, traditionally as caregiver for her children, her parents, and possibly in-laws or extended family members, may lessen her ability to work and may also leave her with no time to participate in the PMTCT program.

### Health system

Fig 3 overlays health system considerations (green) on poverty and gender factor relationships. Service design is shaded to indicate key targets (bolded arrows) for improvement based on this analysis. Fig 3 shows that service design affects service quality—such as through a lack of privacy for clients that may lead to indirect public disclosure of their HIV status. Service design can also affect psychosocial health. For example, in an opt-out system for testing and starting treatment, women cannot always access familial support for decision making while they are at the health facility. Service design can lead to involuntary disclosure and lack of time because of long wait times at the facility and the distance to a facility that offers PMTCT services. It can also directly affect participation in the PMTCT program by discouraging male involvement when service delivery occurs during antenatal clinic visits, which are viewed as women-only services. However, service design can improve knowledge about PMTCT through educational sessions, and could support women to disclose their status to family or friends.

Service quality affects psychosocial health if health workers are rude, clients feel stigmatized, or counseling is inadequate. It also affects disclosure through lack of privacy and lack of time, which can be caused by staff shortages. On the other hand, high quality services could support women to disclose their status. Service quality also directly affects participation in PMTCT services because a perception of low-quality services discourages ongoing participation.

Several factors were sites of confluence or interaction for multiple themes (poverty, gender, or health system), including psychosocial health and lack of time (all three themes); ART use and work (poverty and gender); disclosure (gender and health system), and knowledge about PMTCT (poverty and health system). In addition, our count of incoming arrows indicates that psychosocial health (9), ART use (5), work (4), and no time (4) are affected by the greatest number of other factors. Factors having the greatest number of effects are service design (6), service quality (4), and poverty (4).

Feedback loops were called out in the text describing factor relationships, above. Table 2 lists 66 identified loops. Some of them are simple, with only two variables. Loops become larger as other factors become relevant. Many loops could be reinforcing or balancing, depending on the effect of ART on a woman’s health.

## Discussion

Evidence-based learning should ensure that health-promoting policies succeed, but the ability to document evidence in complex systems is often weak, and the process is slow [14]. The Option B+ approach to PMTCT service provision has been rapidly adopted and some concerns have been raised about women’s participation [11,21]. This analysis documented interactions among the factors that affect women’s participation in PMTCT services under Option B+, using evidence from the literature and our qualitative research conducted in Malawi and Uganda. We deconstructed the challenge of retaining women in PMTCT services into a series of cause-and-effect relationships in order to identify interactions and determine appropriate factors on which to intervene. We created CLDs to define a complex system of interrelationships among poverty, gender, and health system factors. These diagrams revealed a web of effects that may compound disadvantage among HIV-positive women and hinder their participation in services designed to improve their health. Our categorization of factors within three main themes enabled analysis of the particular effects of poverty, gender, and the health system on women’s participation in PMTCT services and facilitated the identification of specific areas for intervention.

### Psychosocial health

The diagrams indicate that the effect of psychosocial health, which we determined through evidence related to self-esteem, self-efficacy, and social support, on women’s participation in PMTCT services cannot be overstated. Because of the number of factors that affect it, psychosocial health is a difficult variable to modify, and it requires particular attention. An intervention that targets just one or a few of its determinants will not succeed because there are several other determinants. Psychosocial health is affected by several factors related to poverty, gender, and the health system and by personal factors that are not included in the diagrams, such as genetics, health history, perception of illness, etc.).

Promising psychosocial health interventions for HIV-positive women include peer support [22] and counseling [23] programs. However, according to our analysis, the success of those interventions would depend in part on their ability to counter negative influences, mainly related to gender and the health system, in order to increase PMTCT participation. Recognizing the various factors that may have a negative impact on a woman’s psychosocial health can lead to a stronger intervention design. For example, a peer support intervention could focus on helping women to address the cycle of disclosure, stigma, and psychosocial health. That would be most effective if it were concurrent with interventions addressing other identified determinants of psychosocial health, such as a change in PMTCT service design and quality to improve privacy protections; outreach to and inclusion of male partners; staffing levels; and health workers’ behavior toward clients. In addition, research suggests that a woman’s perception of her illness influences her decision to participate in PMTCT services [19].

### Balancing loops

In some circumstances a positive action—e.g., ART use or work—might lead to a decrease in PMTCT service participation (balancing loop). Because some women did not want to start ART when they were feeling well [13,19], we hypothesized that if women take ART and begin to feel better, they may be less motivated to continue participating in the program because they no longer feel ill. Moreover, they may stop participating if they experience unpleasant side effects from ART [19]. They may also stop participating when they think the risk for transmission to their infants has passed [24]. Better physical health can lead to more work outside the home, which reduces poverty but also the time available to obtain PMTCT services. These potential negative downstream consequences of ART use or work might be offset by changes in service design to confidentially provide PMTCT services to clients or to arrange service hours convenient for women. Peer support and specialized counseling may help women cope with negative side effects of ART. Services can also be designed to ensure adequate knowledge about PMTCT and the risks and benefits of lifelong ART use among program clients and their family members.

We identified another potentially balancing loop between work and poverty: As work increases, poverty decreases, and as poverty decreases, the need to work decreases. We also identified work as part of a reinforcing loop with decision-making autonomy, hypothesizing that as a woman’s work outside the home increases, her decision-making autonomy also increases. However, given prevailing social norms, we note that a woman’s dependency on her spouse or partner may not change even though she is working [25,26].

### Modifiable factors

We also identified factors that are important to control, given their multitude of effects. Service design and service quality are modifiable factors that influence several others. Given the range of negative experiences reported in the literature [2,13,18], obtaining input from clients about their service experiences and implementing their recommendations to improve service quality could have a positive effect on program participation. In addition, health administrators could facilitate learning about effective service provision between high- and low-performing (in terms of client retention) sites. Service integration, family-centered approaches and lay healthcare providers are service design interventions that could improve retention of PMTCT program participants [27].

CLDs are tools to promote learning in complex systems. Diagramming is one part of an iterative process for solving problems, and CLDs are part of the larger field of systems dynamics modeling [28]. Systems dynamics modeling has been applied to various public health issues over the past several decades, with examples in heart disease [29], substance abuse [30], diabetes [31], and other areas of health research [28]. In the area of HIV/AIDS, systems dynamics modeling has been used to model HIV transmission [32–34]. Ours is the first attempt to model women’s participation in PMTCT services.

This analysis has limitations. We diagrammed causal linkages informed by empirical evidence and hypotheses, but we did not quantify or test this model. Future studies could test it, in whole or in part. Given the complexity we depict, future studies will need to accommodate interactions and feedback loops; to do that, they will need to be longitudinal rather than cross-sectional. We did not account for individual personal factors such as health status or health history, nor did we explicitly explore cultural factors which could affect women’s participation in PMTCT services. This analysis relies mainly on the findings of the parent study, which was conducted in two countries. Although we included findings from literature, this analysis does not represent every factor or linkage related to PMTCT participation globally. In addition, as implementation under Option B+ matures, other factors will emerge to further inform documentation of this complex system. And although not every causal arrow is as important as others, we considered them all equally. Further quantitative and qualitative research could create a ranking for the arrows, which would inform intervention prioritization.

Our causal loop diagrams reveal a complex system of poverty, gender, and health system influences on women’s participation in PMTCT services. We built upon the existing literature to further hypothesize how these influences interact in order to design better interventions. We discussed opportunities for high-impact, concurrent interventions that would most likely be more effective than single interventions in areas that are shown to have limited reverberation throughout the system. This analysis demonstrates both the importance of diagramming causes and effects among factors affecting health service utilization to recognize the potential impact of those relationships beyond their immediate effects, and the utility of causal loop diagrams for understanding interrelationships and documenting the complex system they form. These causal loop diagrams enable practitioners and researchers to consider complexity in their future intervention and research plans.

## Conclusions

The PMTCT participation of HIV-positive women is influenced by a complex interaction of poverty, gender, and health system factors. Psychosocial health is an important factor to monitor, and interventions to improve it should address multiple determinants. PMTCT service design is modifiable at all levels of the health system and is an important factor on which to intervene because it affects several other factors that influence program participation. Flexible approaches to service delivery that meets individual clients’ needs and expectations should be tested.

## References

[1] World Health Organization, United Nations Children’s Fund. Guideline: updates on HIV and infant feeding: the duration of breastfeeding, and support from health services to improve feeding practices among mothers living with HIV. Geneva: World Health Organization; 2016. 27583316

[2] Flax VL, Yourkavitch J, Okello ES, Kadzandira J, Katahoire AR, Munthali AC. “If my husband leaves me, I will go home and suffer, so better cling to him and hide this thing”: The influence of gender on Option B+ prevention of mother-to-child transmission participation in Malawi and Uganda. PLoS ONE 2017;12(6): e0178298 doi: 10.1371/journal.pone.0178298 28594842 10.1371/journal.pone.0178298 PMC5464556

[3] Tweya H, Gugsa S, Hosseinipour M, Speight C, Ng’ambi W, Bokosi M, et al Understanding factors, outcomes and reasons for loss to follow-up among women in Option B+ PMTCT programme in Lilongwe, Malawi. Trop Med Int Health. 2014; 19: 1360–66. doi: 10.1111/tmi.12369 25087778 10.1111/tmi.12369

[4] WHO. Guideline on when to start antiretroviral therapy and on pre-exposure prophylaxis for HIV. Geneva, Switzerland: WHO; 2015. 26598776

[5] Chinkonde JR, Sundby J, Martinson F. The prevention of mother-to-child HIV transmission programme in Lilongwe, Malawi: why do so many women drop out. Reprod Health Matters. 2009;17: 143–51. doi: 10.1016/S0968-8080(09)33440-0 19523591 10.1016/S0968-8080(09)33440-0

[6] Bwirire LD, Fitzgerald M, Zachariah R, Chikafa V, Massaquoi M, Moens M, et al Reasons for loss to follow-up among mothers registered in a prevention-of-mother-to-child transmission program in rural Malawi. Trans R Soc Trop Med Hyg. 2008; 102: 119–200. 10.1016/j.trstmh.2008.04.002 18485431

[7] Clouse K, Schwartz S, Van Rie A, Basset J, Yende N, Pettifor A. “What they wanted was to give birth; nothing else”: barriers to retention in Option B+ HIV care among postpartum women in South Africa. J Acq Immune Defic Syndr. 2014;67: e12–e8. 10.1097/QAI.0000000000000263 PMC6686681 24977376

[8] Hodgson I, Plummer ML, Konopka SN, Colvin CJ, Jonas E, Albertini J, et al A systematic review of individual and contextual factors affecting ART initiation, adherence, and retention for HIV-infected pregnant and postpartum women. PLoS One. 2014;9: e111421 doi: 10.1371/journal.pone.0111421 25372479 10.1371/journal.pone.0111421 PMC4221025

[9] Elwell K. Facilitators and barriers to treatment adherence within PMTCT programs in Malawi. AIDS Care. 2016;28: 971–5. doi: 10.1080/09540121.2016.1153586 26984065 10.1080/09540121.2016.1153586

[10] Kebaabetswe PM. Barriers to participation in the prevention of mother-to-child HIV transmission program in Gaborone, Botswana: a qualitative approach. AIDS Care. 2007;19: 355–60. doi: 10.1080/09540120600942407 17453569 10.1080/09540120600942407

[11] Coutsoudis A, Goga A, Desmond C, Barron P, Black V, Coovadia H. Is Option B+ the best choice? South Afr J HIV Med. 2013;14(1): 8–10.

[12] Flax V, Yourkavitch J, Kadzandira J, Munthali AC. Gender factors influencing participation in the prevention of mother-to-child transmission of HIV Program in Malawi under Option B+. Measure Evaluation publication TR-16-142, 2016.

[13] Yourkavitch J, Flax V, Okello E, Katahoire A. Gender factors influencing participation in the elimination of mother-to-child transmission of HIV Program in Uganda under Option B+. Measure Evaluation publication TR-16-141, 2016.

[14] Sterman JD. Learning from evidence in a complex world. Am J Public Health. 2006;96(3): 505–14. doi: 10.2105/AJPH.2005.066043 16449579 10.2105/AJPH.2005.066043 PMC1470513

[15] Hassmiller Lich K, Frerichs L, Fishbein D, Bobashev G, Pentz MA. Translating research into prevention of high-risk behaviors in the presence of complex systems: definitions and systems frameworks. Transl Behav Med 2016;6: 17–31. doi: 10.1007/s13142-016-0390-z 27012250 10.1007/s13142-016-0390-z PMC4807191

[16] Ngarina M, Tarimo EAM, Naburi H, Kilewo C, Mwanyika-Sando M, Chalamilla G, et al Women’s preferences regarding infant or maternal antiretroviral prophylaxis for prevention of mother-to-child transmission of HIV during breastfeeding and their views on Option B+ in Dar es Salaam, Tanzania. PLoS One 2014;9(1). 10.1371/journal.pone.0085310 PMC3899007 24465532

[17] Gourlay A, Birdthistle I, Mburu G, Iorpenda K, Wringer A. Barriers and facilitating factors to the uptake of antiretroviral drugs for prevention of mother-to-child transmission of HIV in sub-Saharan Africa: A systematic review. J Int AIDS Soc. 2013;16: 1–21. 23870277 10.7448/IAS.16.1.18588 PMC3717402

[18] Kim MH, Zhou A, Mazenga A, Ahmed S, Markham C, Zomba G, et al Why Did I Stop? Barriers and Facilitators to Uptake and Adherence to ART in Option B+ HIV Care in Lilongwe, Malawi. PLoS ONE 2016;11(2): e0149527 doi: 10.1371/journal.pone.0149527 26901563 10.1371/journal.pone.0149527 PMC4762691

[19] Zhou A. The uncertainty of treatment: Women’s use of HIV treatment as prevention in Malawi. Soc Sci Med 2016;158: 52–60. doi: 10.1016/j.socscimed.2016.04.013 27111435 10.1016/j.socscimed.2016.04.013

[20] Duff P, Rubaale T, Kipp W. Married men’s perceptions of barriers for HIV-positive pregnant women accessing highly active antiretroviral therapy in rural Uganda. Int J Womens Health 2012;4: 227–233. doi: 10.2147/IJWH.S31807 22675268 10.2147/IJWH.S31807 PMC3367405

[21] Matheson R, Moses-Burton S, Hsieh A, Dilmitis S, Happy M, Sinyemu E, et al (2015) Fundamental concerns of women living with HIV around the implementation of Option B+. J Int AIDS Soc. 18(Suppl 5): 20286 doi: 10.7448/IAS.18.6.20286 26643459 10.7448/IAS.18.6.20286 PMC4672458

[22] Shroufi A, Mafara E, Saint-Sauveur JF, Taziwa F, Viñoles MC. Mother to mother peer support for women in prevention of mother to child transmission (PMTCT) programmes: a qualitative study. PLoS One 2013;8(6): e64717 doi: 10.1371/journal.pone.0064717 23755137 10.1371/journal.pone.0064717 PMC3673995

[23] Farquhar C, Kiarie JN, Richardson BA, Kabura MN, John FN, Nduati RW, et al Antenatal couple counseling increases uptake of interventions to prevent HIV-1 transmission. J Acq Immune Defic Syndr. 2004;37(5): 1620–6. 10.1097/00126334-200412150-00016 PMC3384734 15577420

[24] Webb R, Cullel M. Understanding the perspectives/experiences of women living with HIV regarding Option B+ in Uganda and Malawi. Amsterdam, Netherlands: Global Network of People Living with HIV. 2013. http://www.gnpplus.net/resources/option-b-understanding-perspectivesexperiences-of-women-living-with-hiv/

[25] Kyomuhendo G, McIntosh M. Women, work and domestic virtue in Uganda, 1900–2003. Cumbria, UK: Long House Publishing Services; 2006.

[26] Otiso KM. Culture and customs of Uganda. Westport, CT, USA: Greenwood Press; 2006.

[27] Vrazo A, Firth J, Amzel A, Sedillo R, Ryan J, Phelps BR. Interventions to significantly improve service uptake and retention of HIV-positive pregnant women and HIV-exposed infants along the prevention of mother-to-chil transmission continuum of care: systematic review. Trop Med Int Health 2017; doi: 10.1111/tmi.13014 29164754 10.1111/tmi.13014

[28] Homer JB, Hirsch GB. System dynamics modeling for public health: background and opportunities. Am J Public Health. 2006;96: 452–458. doi: 10.2105/AJPH.2005.062059 16449591 10.2105/AJPH.2005.062059 PMC1470525

[29] Luginbuhl W, Forsyth B, Hirsch G, Goodman M. Prevention and rehabilitation as a means of cost-containment: the example of myocardial infarction. J Public Health Policy. 1981;2: 1103–1115. 6788801

[30] Homer JB. A system dynamics model of national cocaine prevalence. Syst Dyn Rev. 1993;9: 49–78.

[31] Homer J, Hirsch G, Minniti M, Pierson M. Models for collaboration: how system dynamics helped a community organize cost-effective care for chronic illness. Syst Dyn Rev. 2004;20: 199–222.

[32] Roberts C, Dangerfield B. Modelling the epidemiological consequences of HIV infection and AIDS: a contribution from operational research. J Oper Res Soc. 1990;41: 273–289.

[33] Homer JB, St. Clair CL. A model of HIV transmission through needle sharing. Interfaces. 1991;21: 26–49.

[34] Dangerfield B, Fang Y, Roberts C. Model based scenarios for the epidemiology of HIV/AIDS: the consequences of highly active antiretroviral therapy. Syst Dyn Rev. 2001;17: 119–150.

